# Hospital Contracts and Prices

**Published:** 1910-10-15

**Authors:** 


### HOSPITAL CONTRACTS AND PRICES.

### SURGICAL DRESSINGS.

We have recently been in correspondence with a hospital secretary, who before entering into his three-monthly contacts for dressings was anxious to know where dressings similar to those quoted in our article on "Surgical Dressings," of May 7 last, page 177, could be obtained. The correspondence has brought out one or two interesting points. The list he furnished us with, showing the articles used and the average consumption, contained certain items not generally purchased by large hospitals. One such article is ribbon gauze. We have inquired from a very large dressing manufacturer, and he informs us that though ribbon gauze is supplied to surgeons for use in private practice, he seldom supplies it to hospitals. It is dearer than ordinary gauze, and is a refinement which we think in the circumstances might be dispensed with in hospitals. Surgeons using this article in hospitals might very fairly be asked if they consider ribbon gauze an absolute necessity.

#### Present Prices.

Since the article of May 7 was written there has been a slight rise in the price of dressings, the cotton market having had an upward tendency; in addition to which the continued depression in trade has reduced the output of the waste cotton from which the cheaper dressing wools, etc., are manufactured.

The following list gives a comparison of the prices ruling during the first and second halves of the present year, where six-months' contracts have been arranged.

| | Prices. Jan. 1 to June 30 | Prices. July 1 to Dec. 31 |
|---|---|---|
| Calico, 31 inches ... ... | 2⅜d. per yard | 2⅜d. per yard |
| Bandages, fine, grey and white— | | |
| 2 inches ... ... ... | 5/6 per gross | 6/- per gross |
| 2½ " ... ... ... | 6/10 " | 7/4 " |
| 3 " ... ... ... | 8/1 " | 8/7 " |
| Lint, white ... ... ... | 1/2 per lb. | 1/2 " |
| " boric ... ... ... | 9d. " | 9½d. " |
| Gauze, white ... ... | 5/1½ per 100 yds. | 5/9 per 100 yds. |
| " " plugging ... | 6/6 " " | 7/3 " " |
| " cyanide ... ... | 6/3 " " | 6/3 " " |
| " iodoform ... ... | 11/- " " | 11/- " " |
| Wool, absorbent, white ... | 6d. per lb. | 6½d. per lb. |
| " grey ... ... ... | 4¾d. " | 5d. " |
| Wood-wool wadding ... | 7d. " | 7d. " |
| Surgeons' tow ... ... | 20/- per cwt. | 20/- per cwt. |
| Guttapercha tissue ... | 4/3 per lb. | 5/6 per lb. |
| Jaconet ... ... ... | 1/2 yard | 1/2 yard |

#### Grey v. White O.W. Bandages.

Our correspondent remarks that the opinion in the wards of his institution is that the grey bandage is not as serviceable as the white, and that a sling fixed up with the grey is not so firm and is liable to give. The fact is that a grey bandage should be stiffer than a white if both are woven with the same number of threads to the inch, as the bleaching process necessary to produce the white bandage softens the material. The grey bandage should therefore be more serviceable for slings than the white.

